# Alanine Rewires the Communication Pathways Established During the Allosteric Activation of Liver Pyruvate Kinase by Fructose Bisphosphate

**DOI:** 10.1021/acs.jcim.6c00643

**Published:** 2026-05-26

**Authors:** Jacques Kumutima, Xin-Qiu Yao, Donald Hamelberg

**Affiliations:** † Department of Chemistry, 1373 Georgia State University, Atlanta, Georgia 30302-3965, United States; ‡ Department of Chemistry, 14720 University of Nebraska Omaha, Omaha, Nebraska 68182, United States

## Abstract

Allostery enables enzymes to transmit information from distant ligand-binding sites to catalytic centers through residue-to-residue communication pathways. Human liver pyruvate kinase (hL-PYK) is a prototypical system that integrates activating input from fructose-1,6-bisphosphate (FBP) and inhibitory input from alanine, yet how these opposing signals propagate through the protein remains unclear. Here, we combine multimicrosecond molecular dynamics of the hL-PYK tetramer with residue–residue contact analysis and network/path mapping to define the underlying communication pathways. Contact-change correlations show that alanine antagonizes FBP by reversing many FBP-induced rearrangements. Difference contact network analysis and path enumeration reveal that FBP and alanine rewire information flow along partially overlapping but distinct routes between the allosteric and active sites; residues with high path degeneracy, acting as hubs, emerge as key conduits and include positions that overlap with prior mutational hotspots while also suggesting new sites for experimental testing. Residue community analysis further demonstrates intercommunity edges that switch sign or weaken upon alanine binding, indicating inhibition via residue–residue contact network rerouting. Together, these results support a mechanistic model in which alanine rewires the communication pathways established during FBP-mediated activation of hL-PYK and provide concrete hypotheses for modulating enzyme activity with allosteric therapeutics.

## Graphical abstract

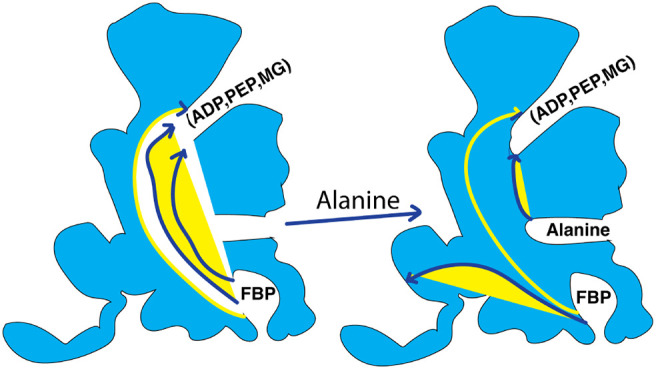

## 1 Introduction

Cells regulate metabolic flux not only by changing enzyme abundance but also by transmitting information through enzymes themselves. Small-molecule effectors bind at sites distant from catalysis and redistribute conformational ensembles,^1^ a phenomenon known as allostery, which allows signals to travel along specific residue-to-residue communication pathways. Different effectors can strengthen, weaken, or even reroute these pathways. Understanding how such signaling is organized and how opposing effectors reconfigure it is essential for explaining metabolic control and for designing allosteric therapeutics. Liver pyruvate kinase (L-PYK) is an ideal model because it integrates activating input from fructose 1,6-bisphosphate (FBP) and inhibitory input from alanine; here, we ask how these metabolites shape and, in the case of alanine, rewire the communication network within human L-PYK.

Kinases catalyze phosphoryl transfer reactions across diverse metabolic pathways in the liver, kidneys, muscles, and red blood cells. Among them, pyruvate kinase (PYK) performs the final step of glycolysis, transferring a phosphate from phosphoenolpyruvate (PEP) to ADP to generate ATP and pyruvate. During catalysis, the active site binds PEP and ADP and coordinates two magnesium ions (Mg^2+^). Because of its central role in glycolysis, dysregulation of PYK activity is linked to tumor proliferation.^2,3^ PYK is a homotetramer that exhibits cooperative behavior among its subunits (Figure 1), and each monomer harbors an active site as well as distinct allosteric binding sites for each regulatory ligand.^4^

**Figure 1:**
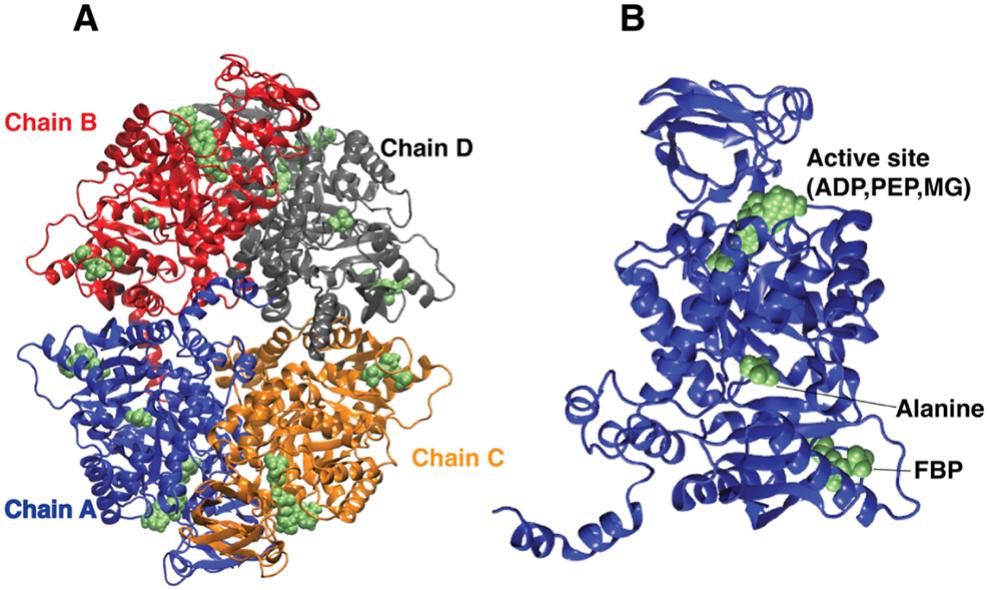
Overall structure of human liver pyruvate kinase (hL-PYK): (A) Tetrameric assembly, with each monomer containing an active site and two allosteric sites (activation and inhibition). Chains are colored A (blue), B (red), C (orange), and D (gray). Ligands are shown as green spheres: ADP, PEP, and Mg^2+^ at the active site; FBP at the activating allosteric site; alanine at the inhibitory site. The structure shown is from the postequilibration MD model (see Methods) from PBD ID 41P7. (B) Enlarged view of a single monomer highlighting the positions of the active and allosteric sites.

Multiple mammalian PYK isoforms exist, including the liver (L) and muscle (M) types.^5^ Here, we focus on human liver pyruvate kinase (hL-PYK), which is expressed primarily in the liver and at lower levels in the kidney. In hepatocytes, hL-PYK functions as a metabolic switch between glycolysis and gluconeogenesis.^6^ The liver and erythrocyte isoforms are encoded by the same gene but are produced under different promoters.^7^ Experimental observations indicate that binding of fructose bisphosphate (FBP; an allosteric activator) perturbs the catalytic site despite an ∼40 Å separation between the FBP and the active site, implying long-range communication within the protein. However, the mechanisms by which conformational changes propagate through hL-PYK remain incompletely defined.

The biomedical relevance of this problem is underscored by the connection between glycolytic control and cancer. Mis-regulation of PYK can contribute to tumorigenesis;^8^ glycolytic metabolites promote tumor proliferation;^9^ many tumors display elevated glycolytic capacity;^10^ and metastatic cells reprogram glucose metabolism and mitochondrial oxidation to support dissemination.^11^ Thus, clarifying how allosteric effectors modulate hL-PYK—FBP as an activator and alanine as an inhibitor—is of both mechanistic and translational interest. What remains unclear is how these opposing signals are transmitted through the protein and which residues are most affected.

To address these questions, we performed molecular dynamics (MD) simulations of the hL-PYK tetramer in the apo state and under multiple ligand-binding combinations (Scheme 1). The simulated tetramer was modeled with complete residues relative to the available PDB structure (PDB ID: 4IP7).^4^ Postsimulation analyses quantified residue–residue contact dynamics within each monomer, identified interaction patterns that change upon ligand binding, and traced allosteric communication pathways using network-based approaches. These analyses reveal residues differentially perturbed by FBP and by alanine and map the routes by which signals propagate (see Results). Notably, FBP activates the kinase while increasing PEP binding,^12^ a behavior we reconcile with the communication pathways inferred from our network analysis. Finally, because the PYK active site is highly charged and competes with natural substrates, direct active-site inhibition poses challenges for small-molecule drug discovery, whereas allosteric modulators offer a promising strategy to tune catalytic activity without blocking substrate binding.^13^

**Scheme 1:**
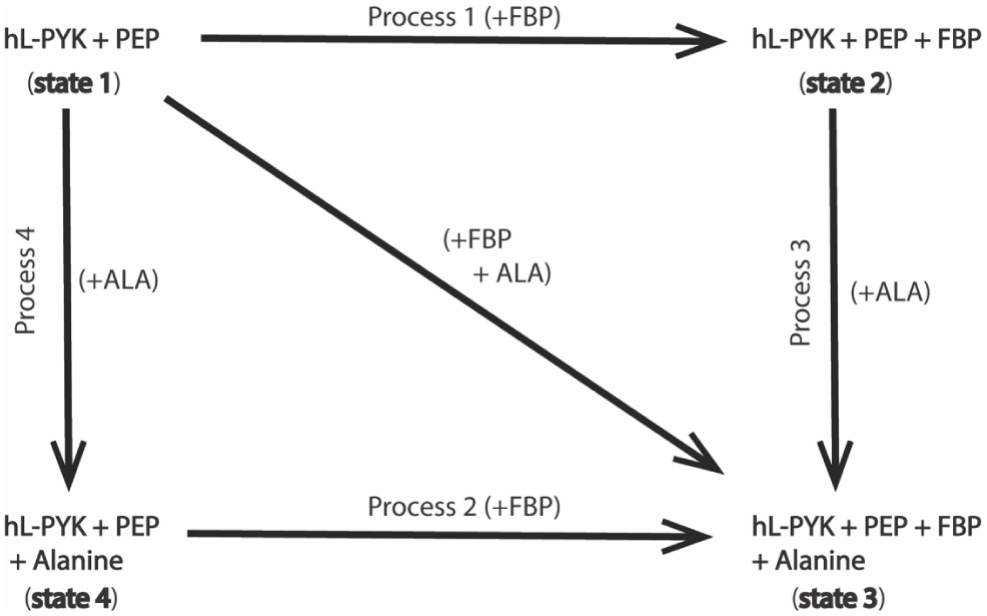
Thermodynamic Cycle for Ligand Binding to hL-PYK^a^

a PEP, ADP, and Mg^2+^ are present at the active site in all states. States are defined as State 1, apo with respect to allosteric ligands; State 2, + FBP; State 3, + FBP+alanine; State 4, + alanine. Processes represent specific binding steps: Process 1, FBP binding in the absence of alanine (State 1→2); Process 2, FBP binding in the presence of alanine (State 4→3); Process 3, alanine binding in the presence of FBP (State 2→3); Process 4, alanine binding in the absence of FBP (State 1→4). Process 5 denotes cobinding of FBP and alanine, considered as a single step

## 2 Materials and Methods

### 2.1 System Preparation

We began with the crystal structure of human liver pyruvate kinase (hL-PYK; PDB ID: 4IP7).^4^ Missing N-terminal residues (1–25) were modeled in MODELER^14^ using the AlphaFold full-length model as a template.^15^ These flexible N-terminal residues are important for protein function.^4^ One hundred models were generated, and the model with the lowest molecular probability density function (molpdf) score was selected for subsequent calculations.

Because PDB 4IP7 lacks several ligands (PEP, ADP, Mg^2+^, and the allosteric inhibitor alanine), additional PYK structures (PDB IDs: 4IMA, 3GR4, 2G50, 4HYV, 2VGB) were used as templates to position ligands in the catalytic and allosteric sites.^4,16–18^ All structures were first superimposed on the selected model using all Cα atoms; coordinates of the relevant ligands were then transferred. In PDB 4IP7, the active site contains citrate rather than PEP/ADP, the allosteric site contains FBP, and Mn^2+^ is present instead of Mg^2+^; ADP is absent. PEP and ADP coordinates were taken from PDB 4IMA; Mg^2+^ from PDB 2VGB; alanine from PDB 2G50; and PDB 4HYV provided an additional PEP-bound reference and Mg^2+^ placement. Two Mg^2+^ ions were placed in each active site (per chain) to stabilize PEP and ADP. Crystal waters were retained in all models.

### 2.2 Molecular Dynamics

Molecular dynamics (MD) simulations were performed with AMBER 22 ^19^ using the ff14SB force field.^20^ Systems were solvated with TIP3P water in a periodic truncated octahedron box extending at least 10 Å from any solute atom; crystallographic waters were retained.^21^ Counterions (Na^+^ /Cl^–^) were added to neutralize each system.

Energy minimization comprised 5,000 steps (3,000 steepest descent followed by 2,000 conjugate gradient) with harmonic positional restraints on solute atoms; the restraint force constant was decreased from 500 to 0 kcal·mol^–1^ ·Å^–2^ over five stages. Systems were heated from 100 to 300 K over 500 ps under NVT conditions using a Langevin thermostat (collision frequency 1.0 ps^–1^) with a 1 fs time step and positional restraints. Five heating stages were used with force constants of 500, 300, 100, 50, and 5 kcal·mol^–1^ ·Å^–2^, respectively. Equilibration was then carried out for 1 ns at 300 K and 1 bar (Monte Carlo barostat, τp = 1.0 ps) with a 2 fs time step and no restraints under NPT. Production simulations were run for 10 μs per system under the same NPT conditions (Table 1), saving conformations every 1 ps. Long-range electrostatics were treated with Particle Mesh Ewald (PME);^22^ a 9 Å real-space cutoff was used for short-range nonbonded interactions. Bonds involving hydrogens were constrained using SHAKE (solute) and SETTLE (water).^23^

**Table 1:** List of MD Simulations

	System	Ligands per chain	Time scale
**1**	hL-PYK	4 PEP,4 ADP, 8 Mg^2+^, 4 K^+^	10 μs
**2**	hL-PYK:FBP	4 PEP,4 ADP, 8 Mg^2+^, 4 K^+^, 4FBP	10 μs
**3**	hL-PYK:Alanine(ALA)	4 PEP,4 ADP, 8 Mg^2+^, 4 K^+^, 4ALA	10 μs
**4**	hL-PYK:FBP:Alanine	4 PEP,4 ADP, 8 Mg^2+^, 4 K^+^4FBP, 4ALA	10 μs

Although simulations were performed in the tetrameric state, trajectory analyses were performed at the monomer level (given the homotetramer’s symmetry). Monomer trajectories extracted from each tetramer simulation were averaged to increase sampling, treating subunits as symmetry-related replicates.

### 2.3 Residue–Residue Contacts and Allosteric Path Analyses

Conformational ensemble changes were quantified via residue–residue contact analysis, which captures both backbone and side-chain reorganization without requiring structural superposition and is sensitive to local rearrangements.^24,25^ A contact was defined for any residue pair whose minimum heavy-atom distance was ≤4.5 Å, provided the residues were separated by at least three positions in sequence.^24,26^ Contacts were computed per frame and converted to contact probabilities over each trajectory. For each ligand-binding process (Scheme 1), state-to-state contact probability differences were calculated, and process-level dynamical correlations were compared. Contact statistics have converged for all simulations (Figure S6).

Difference Contact Network Analysis (dCNA) was performed as described previously.^27,28^ Briefly, in the network, residues were treated as nodes and edges were weighted by the differences in contact strengths between two conformational ensembles (states). Contact strength is defined based on the log-odds of contact probability (*p*) for dynamic contacts (0.1 ≤ *p* ≤ 0.9) or as a constant for nondynamic contacts (−2.0 kcal/mol if *p* > 0.9 and 2.0 kcal/mol if *p* < 0.1). Five dCNA networks were constructed corresponding to the ligand-binding processes in hL-PYK (Scheme 1). In the path analysis, end points were defined at the PEP (active-site) region and the FBP allosteric site; depending on the process, one served as the source and the other as the sink.^29^ For systems lacking a physical ligand at an end point (e.g., FBP-absent or alanine-absent conditions), a dummy node was placed at the corresponding pocket following established procedures.^27,28^ Briefly, pocket residues were first identified as those within 4 Å of the effector (based on the corresponding effector-bound structure). A dummy node was then connected to these pocket residues with equally weighted edges (assigned arbitrary weight values). Note that the exact choice of weight for dummy node-associated edges does not affect the path analysis. We evaluated 5,000 optimal and suboptimal paths per network.

Alternative graph-theoretic approaches of community analysis were considered to aid interpretation of MD data and to map allosteric communications.^30^ Briefly, residue communities were detected from a consensus contact network built using simulations of Processes 1 and 3. Intercommunity contact changes were then evaluated for Processes 1 and 3 (see Scheme 1) by summing up all residue-wise contact changes between communities. The community analysis provides a simplified representation of regional conformational changes and informs how these changes are connected to mediate the allosteric communication between different regions of the protein.

### 2.4 Software

Trajectories were processed with CPPTRAJ from AMBER.^19^ Network analyses were conducted using the Bio3D library with additional in-house scripts.^31–33^ Molecular graphics were rendered in VMD.^34^ Plots were generated in R (ggplot2),^35^ and figures were assembled in Adobe Illustrator (2025 release).

## 3 Results and Discussion

### 3.1 Different Ligand-Binding Processes Are Correlated

Residue–residue contacts were computed from MD snapshots, and contact statistics were evaluated as described previously.^26^ We defined the contact change for a given “process” (a specific ligand-binding transition; see Scheme 1 and Figure 1) as the difference in contact probabilities between the ligand-free state and the corresponding ligand-bound state. Pairwise comparisons of these contact-change profiles across processes yielded correlations that are positive (similar patterns of contact formation/breakage), negative (opposing patterns), or near zero (uncorrelated) (Figure 2). For completeness, we also report “class 0” cases in which a contact changes in one process but is absent or unchanged in the other, reflecting an edge present only in one network.

**Figure 2:**
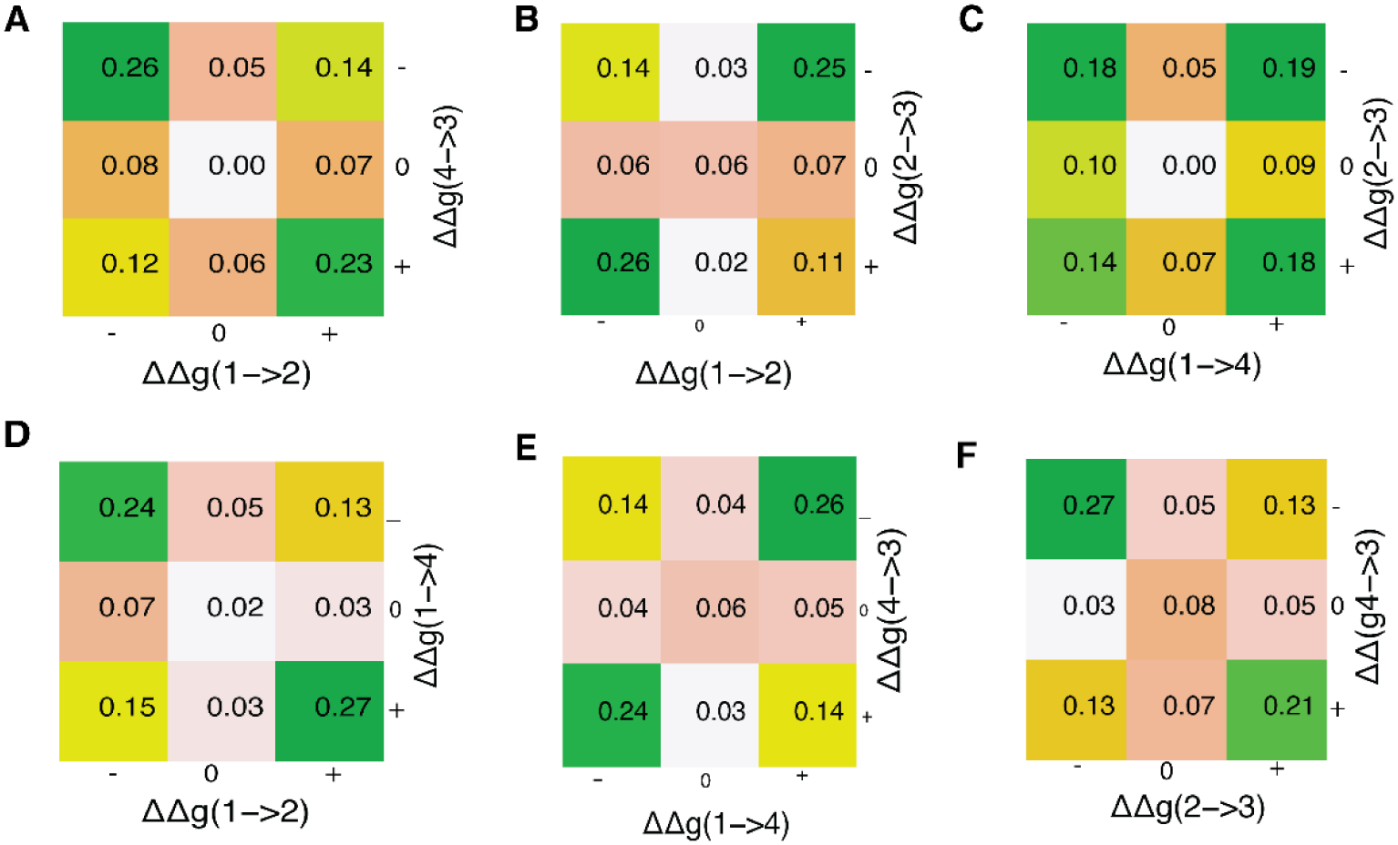
Correlations of residue–residue contact changes across ligand-binding processes: Contacts were taken from the union of network edges across all processes (Scheme 1). For each process, contact changes were computed as differences in contact probabilities between ligand-free and ligand-bound states; pairwise comparisons yield joint sign classifications: negative (−), zero (0), or positive (+). Heat maps encode the joint probabilities, with values labeled in each cell. (A) Process 1 vs Process 3 (FBP binding vs alanine binding on the FBP-bound enzyme): overall positive correlation (mutual information, MI = 0.08 bit), indicating antagonism. (B) Process 1 vs Process 2 (FBP binding with vs without alanine present): overall negative correlation (MI = 0.13 bit), indicating similar contact reorganization by FBP in both backgrounds. (C) Process 4 vs Process 1: correlation as indicated (see color scale), weak/near-zero correlation (MI = 0.06 bit), implying alanine engages distinct residue sets depending on FBP. (D) Process 1 vs Process 4: overall positive correlation (MI = 0.07 bit; contacts similarly affected by FBP binding and alanine binding in the apo background). (E) Process 4 vs Process 3 (MI = 0.11 bit) (F) Process 3 vs Process 2: overall positive correlation (MI = 0.12 bit).

These correlations indicate that allosteric communication involves concerted residue dynamics that depend on the bound effector. The scatter plot (Figure S1) shows details of the respective negative and positive correlations. Some hL-PYK residues are selectively impacted by FBP, others by alanine, and a subset remains relatively insensitive to either ligand. Consequently, the overall contact-change statistics are ligand-specific, consistent with distinct allosteric mechanisms for activation (FBP) and inhibition (alanine).

Figure 2A shows a positive correlation when comparing FBP binding with and without alanine already present, implying that FBP perturbs many of the same contacts regardless of alanine, albeit often with altered magnitudes. In contrast, Figure 2C illustrates a case of weak correlation: alanine binding in the absence of FBP versus alanine binding to the FBP-bound enzyme affects partly distinct residue sets. In Figure 2B, the contact-change profile for FBP binding is negatively correlated with that for alanine binding, indicating that alanine tends to reverse FBP-induced rearrangements. A similar trend is shown in Figure 2E, which compares contact changes during alanine binding and the subsequent FBP binding in the presence of alanine. Additionally, both Figure 2D (comparing FBP and alanine binding in the absence of the other effector) and Figure 2F (comparing FBP and alanine binding in the presence of the other effector) reveal positive correlation, further supporting that the two effectors modulate similar sets of residue contacts. Together, these comparisons support the view that alanine antagonizes FBP’s effect while also introducing its own communication pattern.

### 3.2 Allosteric Communication Mapped by Path Analysis

#### 3.2.1 Paths across the Protein Reveal How Signals Propagate

We used Protein Contact Network (PCN)-based path analysis to identify residues that mediate long-range communication.^27^ In this framework, residues are nodes, and edges are weighted by differences in contact probabilities (converted to free-energy differences) between two states (dCNA).^27,28^ We constructed networks for the ligand-binding processes defined in Scheme 1 and enumerated 5,000 optimal and suboptimal paths between the FBP allosteric site and the active-site PEP region (source/sink as indicated in Figure 3).^29^ “Degeneracy” denotes the fraction of enumerated paths that pass through a given residue. Node degeneracy was also computed using 1,000 and 10,000 paths to evaluate the convergence of results (Figure S5). The degeneracy profiles remain consistent for different numbers of paths, indicating robust identification of residues involved in the allosteric pathways of inhibition and activation.

**Figure 3:**
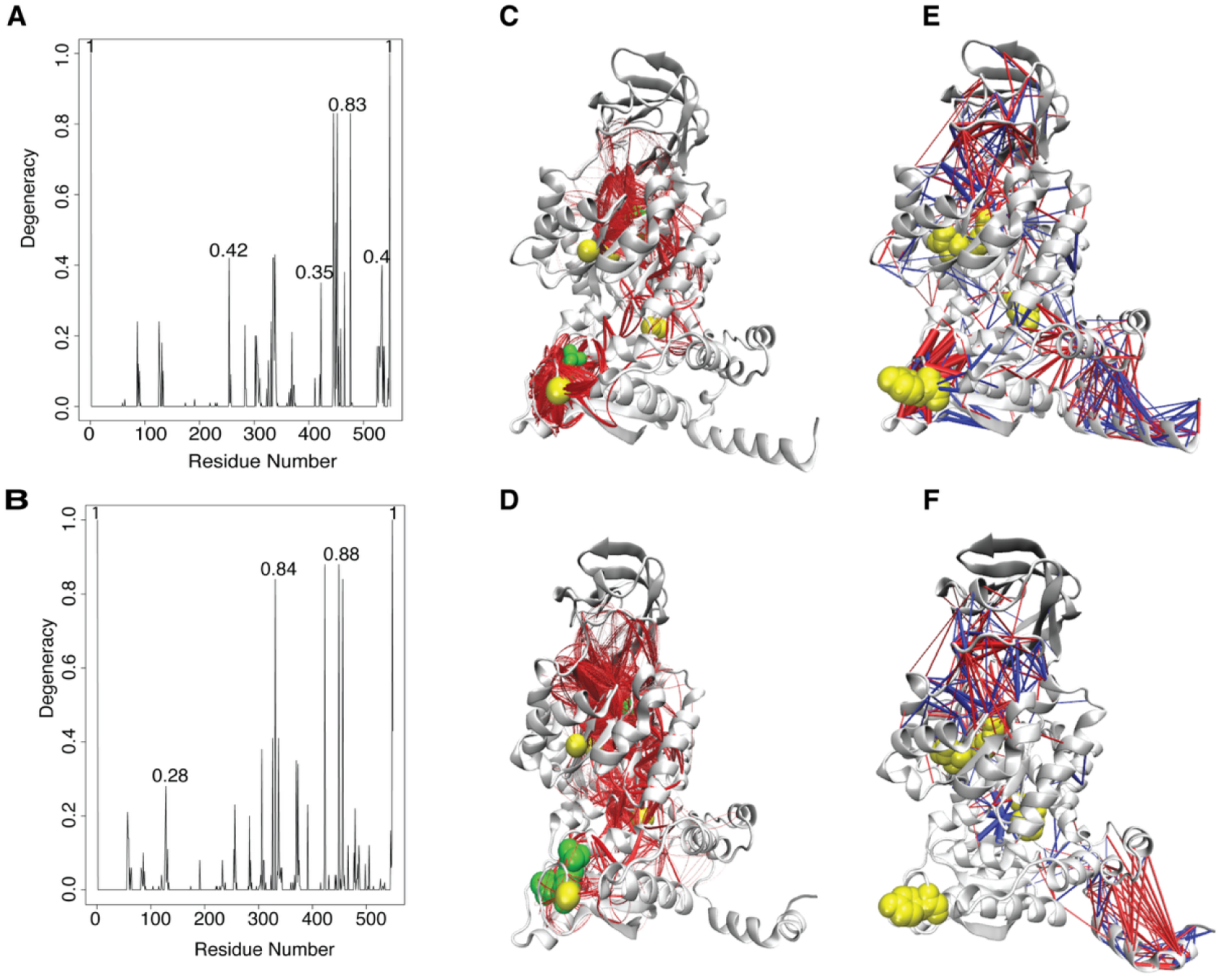
Allosteric paths and contact-network reorganization: (A, B) Node degeneracies (fraction of 5,000 enumerated paths traversing a residue) for Process 1 (FBP binding; State 1→2) and Process 3 (alanine binding on the FBP-bound background; State 2→3). Alanine reroutes several high-degeneracy nodes established by FBP. (C, D) Allosteric path ensembles for Process 1 and Process 3, respectively. Source and sink positions (green) are placed at the PEP region and the FBP site. Red lines trace the 5,000 optimal/suboptimal paths obtained from dCNA networks. Yellow highlights mark residues previously shown to affect activity when mutated. (E, F) Residue–residue contact changes for Process 1 and Process 3. Blue edges denote contact formation; red edges denote contact breakage relative to the prebinding reference. Alanine reverses many FBP-induced edges and introduces new connections centrally.

Comparing the biologically central processes—FBP-driven activation (process 1) and alanine-driven inhibition in the FBP-bound background (process 3)—reveals clear rerouting (Figure 3A,B). Residues 85 (degeneracy 0.24), 252 (0.42), and 533 (0.40) display higher degeneracies in Process 1 than in Process 3, identifying them as preferential conduits of FBP-mediated activation. Notably, residue 85, neighboring residue Asn82, and residue 533, neighboring residue Thr534, were previously implicated by mutation studies in pyruvate kinase function.^36,37^ Conversely, residue 56 (degeneracy 0.21) emerges specifically in Process 3, suggesting a role in alanine-mediated inhibition; this is consistent with mutational sensitivity reported for Arg55/Ser56.^37^ The region around Ser437 carries appreciable degeneracy in both processes and has been linked to FBP responsiveness in PKM2.^38^

The overall “strength” of the allosteric signal can also be summarized by path lengths (Supporting Information - Figure S2). Shorter paths indicate larger associated contact changes, implying stronger allosteric coupling. Although FBP and related analogs form ionic interactions with residues at their binding site,^39^ our network analysis is agnostic to interaction type and instead captures state-dependent communication. As visualized in Figure 3C,D, FBP binding favors one set of routes, whereas adding alanine redirects traffic through alternative residues—consistent with alanine’s capacity to counteract FBP.^40^

#### 3.2.2 Ligand Binding Induces Contact Formation and Breakage

Direct inspection of contact networks highlights where edges form or disappear upon ligand binding (Figure 3E,F). Each effector produces a characteristic pattern: in Process 1 (FBP), several edges near the allosteric site and along the canonical communication corridor are formed (blue), whereas in Process 3 (alanine added to the FBP-bound enzyme) many of these edges are weakened or broken (red), and new edges appear near the center of the monomer, indicating rerouting. Because edges are the carriers of residue-to-residue signals, these changes explain how alanine rewires FBP-initiated communication.^37^

### 3.3 Alanine’s Inhibitory Effect Reverses FBP-Induced Residue-Community Contacts

To examine these effects on a mesoscopic scale, we partitioned the protein into communities based on modularity (10 communities shown in Figure 4). Communities group residues with similar contact-change behavior and often correspond to structural/functional regions. The number of communities was determined by maximizing the modularity of the network (Figure S3). Ligands are shown as separate communities to distinguish ligand-specific effects from protein-intrinsic effects.

**Figure 4:**
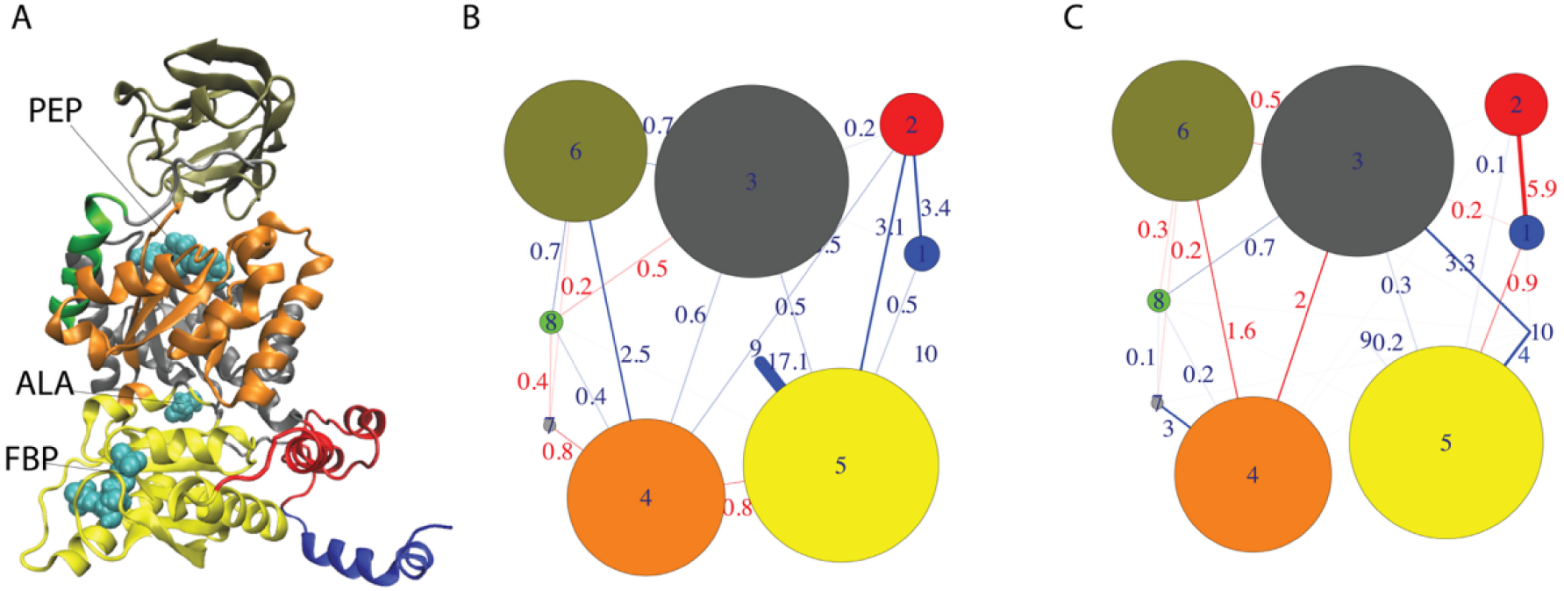
Community-level analysis of contact reorganization: (A) Community partitions for Process 1 and Process 3 (10 communities; ligands shown as separate communities: PEP, Community 8; FBP, Community 9; ALA, Community 10) mapped onto the structure. (B, C) Community-difference networks obtained by mapping the partitions in (A) onto intercommunity contact changes. Several edges that are blue (formation) in Process 1 become red (breakage) in Process 3, consistent with alanine reversing FBP-driven coupling. Node (community) size scales with the number of residues. Edge thickness is proportional to the summed contact-probability change between communities (∑Δp); blue/red edges and labels indicate positive/negative net change.

Across processes, multiple intercommunity edges flip sign. For example, the edge between Community 1 and Community 2 is formed in process 1 (blue) but broken in process 3 (red); the same reversal is observed for the edges between Community 1 and 5 and those between Community 3 and 4. The Community 4–6 edge also switches from blue (process 1) to red (process 3). Note that Communities 3, 4, and 6 are all responsible for substrate binding. By contrast, Community 4–7 reverses from red (process 1) to blue (process 3), indicating compensatory coupling under inhibition. The Community 5–9 edge remains blue in both processes but is thinner in process 3 (with net contact probability change of 17.1 versus 0.2), consistent with reduced contact formation probability under alanine. Community 10 corresponds to the alanine ligand: it is absent (isolated) in process 1 and appears as its own community only in process 3, where alanine is present.

Mapping communities onto the structure (Figure 4A) shows that several partitions are adjacent to the allosteric and active sites. Communities 4 and 5 lie adjacent to FBP and alanine and are therefore most directly influenced by effector binding. Communities 1 and 2 are more distal and flexible; their connecting/disconnecting behavior suggests a role in accommodating long-range conformational shifts. While our analysis nominates these communities as key communication units, the specific biological roles of each partition require experimental testing.

## 4 Conclusions

We used multiple long-timescale MD simulations (10 μs per system) to define how the allosteric activator fructose-1,6-bisphosphate (FBP) and the inhibitor alanine reprogram communication within human liver pyruvate kinase (hL-PYK). The convergence of simulations was verified using Root-Mean-Square Deviation for backbone Cartesian coordinates and residue–residue contact statistics (see Supporting Information S4 and S6). By integrating residue–residue contact analysis, correlations of contact-change profiles, and network/path analyses, we show that alanine rewires many of the FBP-established routes linking the FBP site to the active-site PEP region and introduces alternative pathways. Thus, ligand binding perturbs a distributed network that couples the allosteric and catalytic sites.

Path-degeneracy maps highlight residues that disproportionately carry signal; several coincide with positions previously shown to affect PYK function in mutational studies, and newly predicted key residues provide testable hypotheses for experiments.^36–38^ Community-level analyses further reveal multiple intercommunity couplings that switch sign or weaken when alanine is present, consistent with inhibition via rerouting rather than direct active-site blockade. Although pyruvate kinase subunits function cooperatively, this study focused on the allosteric pathways inside one subunit, and the simulation of all subunits was assumed to capture average ensembles. We also emphasize that our analysis evaluates ensemble-averaged statistics under each effector-binding condition and therefore does not explicitly resolve the potential coexistence of active and inactive conformational states within a given condition. As a result, we do not assess the relative stability of these states, how their populations shift upon effector binding, or the transition pathways among them.

Together, these findings support a mechanistic model in which alanine counteracts FBP-mediated activation by rewiring the protein’s communication pathways, thereby diminishing catalytic competence through network-level effects. Given the links between PYK regulation and cancer, mapping these pathways can inform the design of allosteric modulators that tune activity without competing with substrates.

## Supplementary Material





## Data Availability

MD input files are available as an attachment to the Supporting Information. Residue–residue contact analysis, community analysis, and path analysis were performed using codes available here: https://github.com/The-Hamelberg-Group/dcna.

## Notes

The correlation of residue contact changes during different effector binding processes (Figure S1). Comparison of path length distributions (Figure S2). The modularity score used to determine the number of communities (Figure S3). Root mean square deviation confirms the convergence of the simulations (Figure S4). Node degeneracies for varying numbers of paths in process 3 (Figure S5). The convergence of simulations with respect to contact statistics (Figure S6) (PDF)
MD input files (ZIP)
