# Supplementary material for: Alanine Rewires the Communication Pathways Established During the Allosteric Activation of Liver Pyruvate Kinase by Fructose Bisphosphate
Source: J Chem Inf Model. 2026 May 26;66(11):6637–43. doi: 10.1021/acs.jcim.6c00643 (PMC13250992; doi:10.1021/acs.jcim.6c00643)
Supplement: Supplementary file 1 [file ci6c00643_si_001.pdf]

Supporting Information

Alanine rewires the communication pathways established during the allosteric activation of liver pyruvate kinase by fructose bisphosphate

Jacques Kumutima[1], Xin-Qiu Yao[2], Donald Hamelberg*[1]

[1]Department of Chemistry, Georgia State University, Atlanta, Georgia 30302-3965, USA.

[2]Department of Chemistry, University of Nebraska Omaha, Omaha, Nebraska 68182, USA.

*Corresponding Author Tel.: (404) 413-5564; E-mail: dhamelberg@gsu.edu

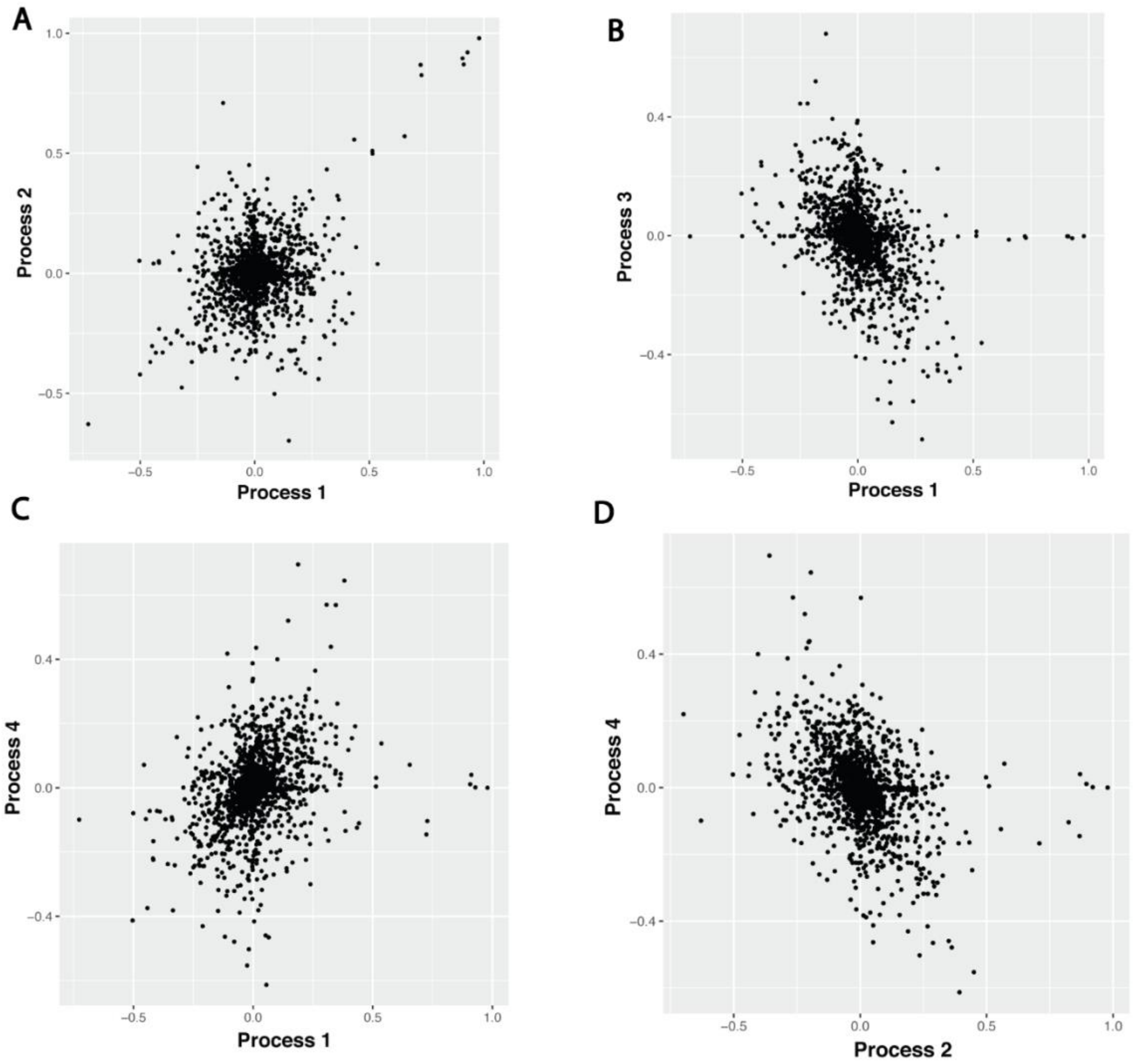

Figure S1. The correlation of residue contact changes during different effector binding processes.

A-positive correlation between process1 and process2. Effect of FBP is positively correlated to the effect of FBP when alanine is present. **B**-negative correlation. Effect of FBP is reversed by the addition of alanine. **C**-positive correlation between process1 and process4. The effect of FPB is negatively correlated to the effect of alanine. **D**-negative correlation. FBP does not reverse the activity of alanine. Some residues are conserved, and they are not always affected by the change. Some residues are also specific to PEP. Some other residues are specific to FBP especially the sparsest points.

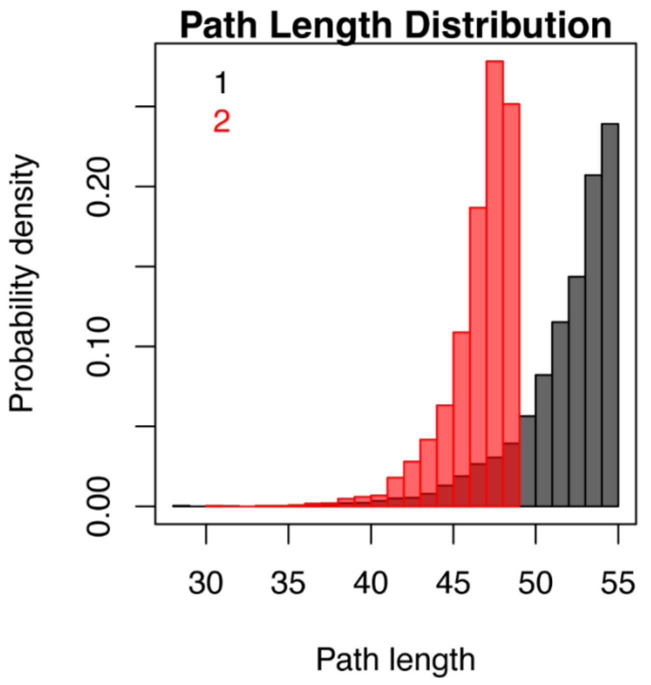

Figure S2. Comparison of path length distributions. (1-black) – Process 1 and (2-red) – Process 3. The peaks show the length of most paths for each network. Process 1 network has relatively longer paths than process 3.

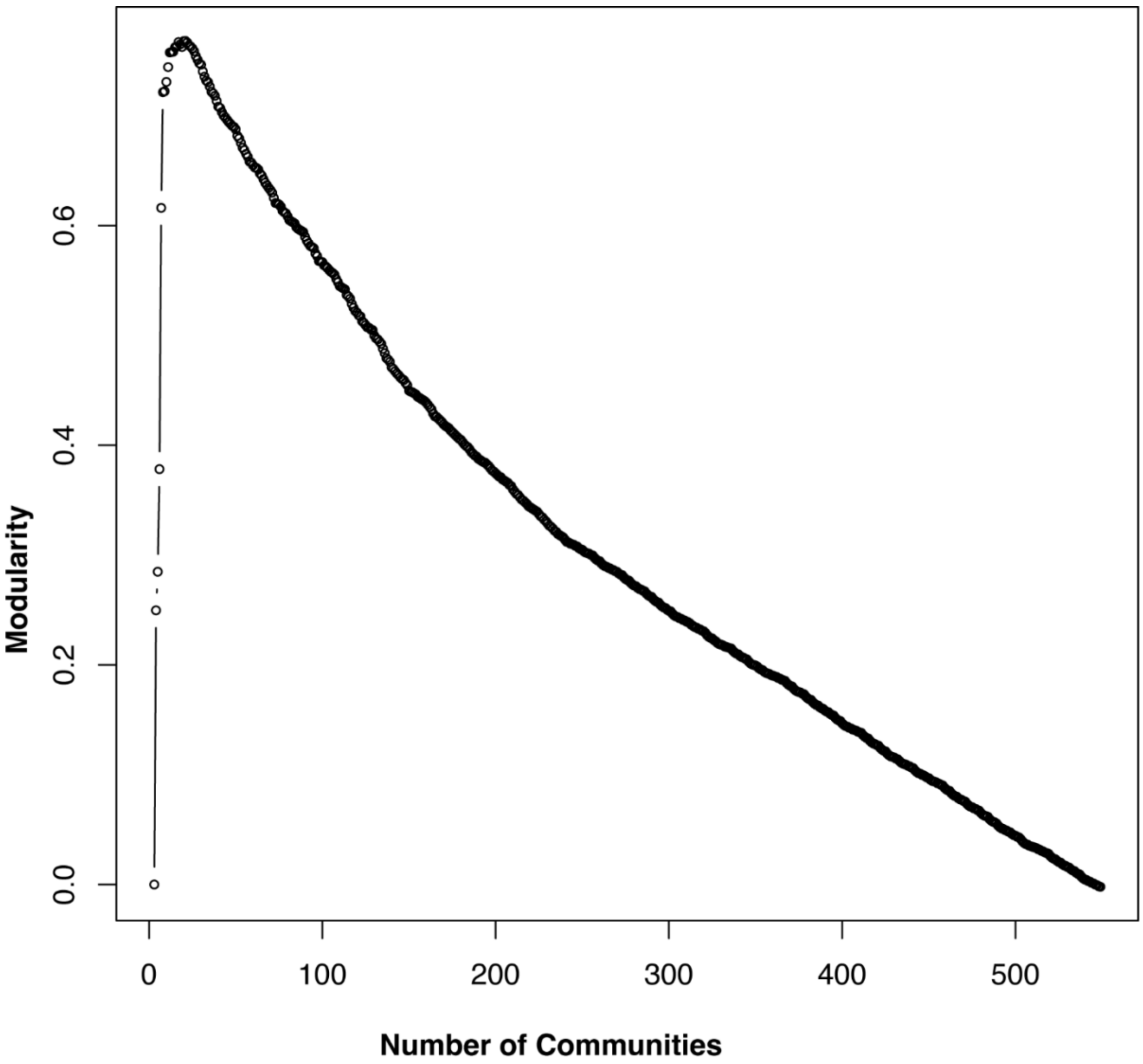

Figure S3. The modularity score to determine the number of communities. The 10 communities that were selected represent the maximum modularity.

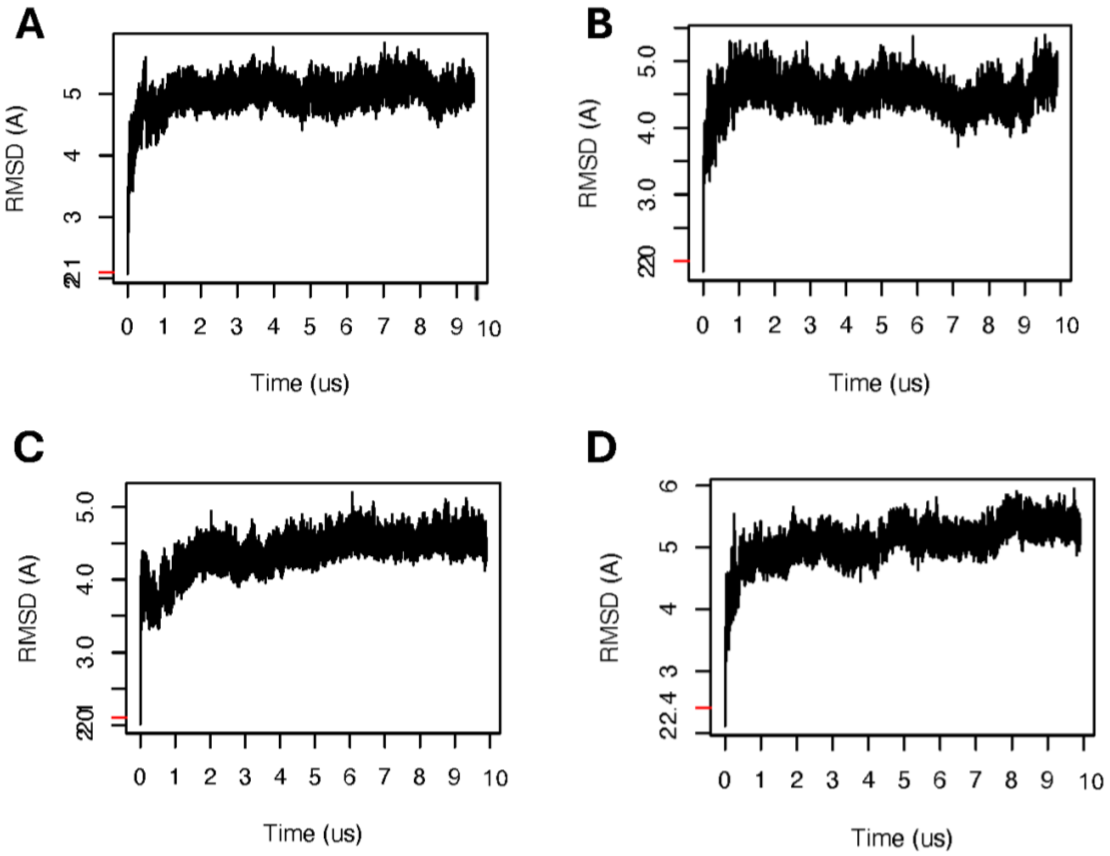

Figure S4. Root mean square deviation confirms the convergence of the simulations. **A**= hlpyk_Pep, **B**= hlpyk_pep_fbp, **C** = hlpyk_pep_fbp_ala, and **D** = hlpyk_pep_ala. The red ticks represent the RMSD for the first frames.

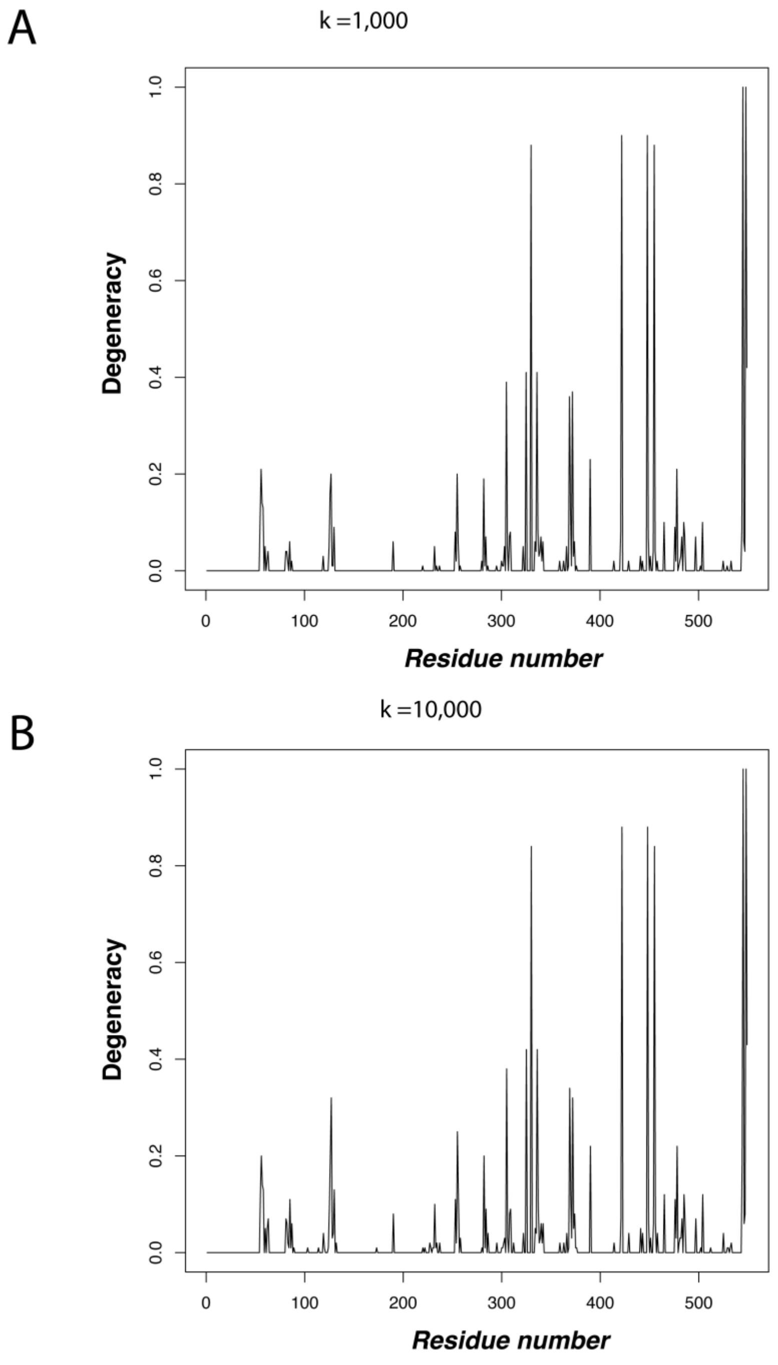

Figure S5. Node degeneracies (tbl = degeneracy, index = residue numbers) for varying numbers of paths in process 3 (alanine binding on the FBP-bound background; State 2→3). Increasing the number of paths from 5,000 to 10,000, or decreasing it to 1,000, did not alter the reported degeneracy peaks in either location or magnitude.

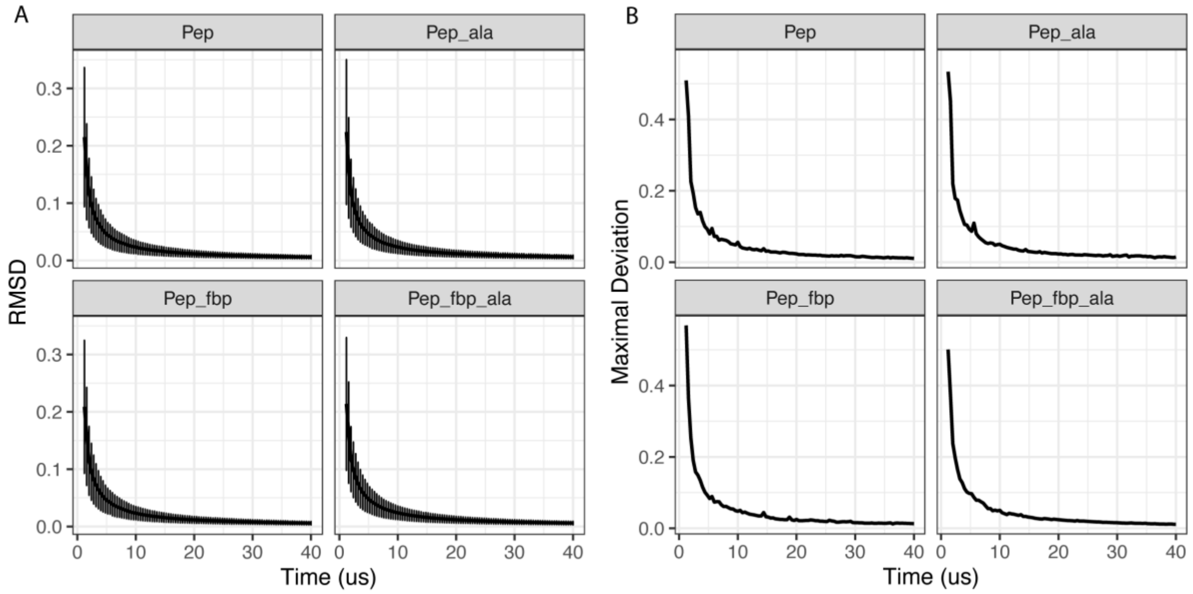

Figure S6The convergence of simulations with respect to contact statistics. A) root mean square deviation of contact probabilities for all dynamic contacts (0.1 ≤ contact probability ≤ 0.9). Error bars are the standard deviations. B) Maximal deviation of contact probabilities.
